# News from Societies

**Published:** 1954-10

**Authors:** 


### *News from Societies*

### COLLEGE OF GENERAL PRACTITIONERS
### SOUTH WEST OF ENGLAND FACULTY

In its first year the Board of the Faculty has concerned itself with establishing general practitioner research and with post-graduate study. Dr. Mallett, the member for research, has put in much time. A Faculty Advisory Panel has been formed, with Professor Milnes-Walker and Dr. A. H. Gale from Bristol University. After much preliminary work by Dr. Mallett of Wimborne, Dr. Hickson of Chippenham, and Dr. Cookson of Hucclecote, Glos., an Obstetric Survey of the Region was started on 1st June. This will run for a year. Dr. Mallett has also devised, with the help of Mr. John Dodd, Hon. Assistant Secretary, a system of quick warnings to members on the research register of any epidemics in or approaching the Region.

Post-graduate study has made a good start. From the founding of the faculty Dr. A. H. Gale, Director of Post-graduate Studies, University of Bristol, has given us every assistance. He is a co-opted member of the Faculty Board. With Dr. Jackson of Crediton he organized a most successful weekend course at Exeter. The recent week's residential course held in Bristol has been equally successful. It is hoped to hold regular weekend and week courses in future. The Faculty is represented on the Post-Graduate Advisory Committee.

*Undergraduate Education.* Dr. Hickson of Chippenham is the member of the Board in charge of this. Bristol University have already started a two-week training in General Practice for final year students, and two lectures in General Practice are given each year. The two-week course seemed to work very well. From the General Practitioners' point of view March and April are somewhat busy months, and for the students it is perhaps too near the Finals for them to give their undivided attention.

It is hoped that the membership will continue to increase. In the first year we have reached 221. It is a good start, but we would like to see many more join us.

D. M. CAMERON,
*Hon. Sec.*

### BRISTOL MEDICO-CHIRURGICAL SOCIETY, 1953–54

The yearly work of this society has now acquired a pattern, almost a rhythm, for which the secretary at least is grateful. Annual General Meeting and Presidential Address are followed by a Clinical Meeting in November. The organizers of this do a great deal of hard work, and the staff of the hospital concerned co-operate nobly to produce an instructive yet delightfully informal evening. In recent years a custom has arisen of a discussion of selected cases following the demonstration of the patients.

The rest of the year's meetings are concerned with scientific papers and again by custom two of these are read by guests, one of whom is invited personally by the president. This session the A.G.M. and clinical meeting have already been reported in the *Journal*, and the Presidential address is to be published soon. Mention has also been made of Mr. Cooke's paper in December.

Our two guests were Sir George McRobert (presidential) and Dr. G. F. Abercrombie (society). The former gave a masterly address at the February meeting on Tropical Medicine, while in April the second delighted us with a philosophical discourse on General Practice.

In January there was an excellent talk by Mr. FitzGibbon on plastic surgery—beautifully illustrated of course. In March Dr. Beryl Corner and Dr. Campbell combined forces to open a discussion on meningitis. Members were left secure in the knowledge that Bristol is the best place in which to suffer from this disease.

In May we had a stroke of good fortune. The visit to Britain of the American College of Surgeons brought several distinguished men, and thanks to the persuasive powers of Professor

Milnes Walker we were able to listen to Dr. Frederick Coller of Ann Arbor, Michigan, on early diagnosis in carcinoma of colon, a masterly talk by a master, simply preaching the simple virtues, and surprising to some from an American, praising bedside examination and history above all else.

BRIAN McCONNELL, *Secretary.*

OFFICERS FOR SESSION 1954–55

*President:* Dr. Gordon Heron. *Treasurer:* Dr. J. MacRae. *Secretary:* Dr. B. E. McConnell.

PROGRAMME

(*Second Wednesday of the month at* 8.15 *p.m.*)

October 13th: General Meeting. **Presidental Address:** Dr. Gordon Heron.

November 10th: Clinical Meeting at Bristol General Hospital.

December 8th: **"Bladder Neck Obstruction":** Mr. Ashton Miller and Mr. J. P. Mitchell.

January 12th: **Report from America:** Dr. J. Cates, Dr. J. Cosh, Mr. J. Peacock and Mr. M. G. Wilson.

February 9th: Dr. McDonald Critchley.

### CLINICAL SOCIETY OF BATH, 1953–54

Seven meetings were held during the session and the following papers were given:

In October 1953 Mr. J. Bastow discussed "**Arthroplasty of the Hip**" with a demonstration of cases. This was followed by a colour film prepared in Bath by Mr. A. E. Burton showing the stages in the cup arthroplasty.

In November, Dr. J. O'Neill read a paper on "**Generalized Lymphadenopathy**" and quoted a series of 100 cases which presented a picture somewhat similar to infectious mononucleosis. This paper was then discussed.

At the December meeting, Mr. B. Blair discussed the effects of "**Hexathide in Toxaemia of Pregnancy,**" with special reference to the pre-eclamptic state.

At the January meeting this year the Society welcomed Professor Milnes Walker who gave a most interesting talk on "**Cardiac Surgery**" which was illustrated by two films and very greatly appreciated.

At the February meeting the speaker, Mr. T. Tizzard, was seriously and unexpectedly indisposed on the morning of the meeting and, as a result, short papers were given on the following subjects:—

Dr. D. Pugh: "**Nephrotic Oedema treated by Acupuncture.**"

Mr. P. Sames: "**Surgery of the Rectum.**"

Dr. G. Kersley: "**The value of deep X-ray therapy in Arthritic Conditions.**"

Dr. J. Bolton: "**Peptic Ulcer in the Aged.**"

Mr. S. Glaser: "**The Preservation of the Hydronephrotic Kidney.**"

In March the Society was addressed by Dr. John Hunt, the Secretary of the College of General Practitioners, on the Development and Aims of the College and present trends in general practice in this country. His paper was most warmly received and excited prolonged discussion.

At the seventh and last Meeting a round-table discussion on "**The Use and Abuse of Antibiotics**" was most usefully developed from the point of view of the General Practitioner, the General Surgeon, and the Pathologist.

THE OFFICERS OF THE SOCIETY 1954–55

*President:* Dr. J. R. O'Neill.

*Future President:* Mr. J. Bastow.

*Committee:* Dr. L. D. Brice; Mr. S. Glaser; Dr. G. D. Kersley.

Meetings will be held on the second Friday of every month from October until April and the October speaker will be Mr. J. M. Sanson who will discuss "**Some quoted Midwifery**".

### DEVON AND EXETER MEDICO-CHIRURGICAL SOCIETY, 1953–54

The Society had a successful year, its last meeting being in April. It has a membership of 194 and the average attendance at ordinary meetings is between 30 and 40.

There were four Clinical meetings preceded by quarter-hour talks on subjects of current interest such as "Some Assorted Skin Conditions"—Dr. John Simpson; "The Role of Fluoride in the Prevention of Dental Decay"—Mr. Jeffrey Fletcher; "Perirenal Pneumograms"—Mr.

Alan Gairdner; "Dysphagia Associated with Lung Complications"—Dr. C. J. Fuller. Two Clinico-pathological Demonstrations were held. There was a Symposium on "Cortisone and A.C.T.H." to which the contributors were Dr. C. J. Fuller, Dr. J. R. Simpson and Dr. J. O. P. Edgcumbe. Mr. Philip Scott gave a lecture on the "Fenestration Operation".

Mr. Norman Capener gave his Presidential Address on March 18th and to it were invited the wives of members, the Dean of Exeter and Mrs. Wallace, and other distinguished persons. There were 140 people present and there was on view a representative display of drawings, hospital minutes, correspondence and portraits of members of the medical staff of the hospital in the eighteenth century.

Mr. Capener spoke on "The Hunterian Influence in Exeter". He referred to the early provision made for the sick of the city up to the foundation of the hospital in 1741 and told the company something of such distinguished early members of the honorary medical staff as Dr. Thomas Glass, Dr. Patch, Dr. Blackhall and Dr. Lee Dicker and of the painter Opie who, like Turner and Reynolds, was a West Country man.

He traced the relationships as teacher and pupil of Cullen, William and John Hunter, Astley Cooper, Abernethy, Charles Bell and our own John Sheldon.

Mr. Capener showed on the screen a number of beautiful portraits including that of John Haddy James about whom there is much biographical material and who served as medical officer to the Guards Regiment at the battle of Waterloo. A relation, Miss Farringdon of Lympstone, was a guest at the meeting, thus completing our link with the past. Mr. Capener showed a number of treasures from the Cathedral Library including first editions of Harvey, Booerhaave and John Hunter, and others from the fine collection in the Hospital Medical Library.

### Officers for Session 1954–55

*President:* Mr. Norman Capener.

*Treasurer:* Dr. G. Stewart Smith.

*Secretary:* Dr. Charles Seward.

### Programme

*(All meetings are held on Thursdays in the Library of the Royal Devon and Exeter Hospital unless otherwise stated.)*

October 28th, 3.30 p.m.: Mr. Osmond-Clarke, C.B.E., F.R.C.S.: **"Pain in the Neck and Arm."** (South Western Dinner.)

November 4th, 3 p.m. (Clinical Meeting): Dr. R. B. H. Tierney, M.D.: **"Differential Diagnosis of Jaundice."**

November 18th, 8.30 p.m.: Clinico-pathological Demonstration.

December 16th, 8.30 p.m.: Dr. F. S. W. Brimblecombe, M.D., M.R.C.P.: **"Some Common Disorders of Childhood."**

January 20th, 3 p.m. (Clinical Meeting): Mr. Philip Scott, F.R.C.S.: **"Diseases of the Ear, Nose and Throat"** (with film).

February 17th, 8.30 p.m.: Clinico-pathological Demonstration.

March 17th, 8.30 p.m.: Dr. J. O. P. Edgcumbe: **"Miscellaneous Haematology."**

April 14th, 8.30 p.m.: Dr. Ffrangçon Roberts, M.A., N.D., F.F.R. (Title awaited.)

April 28th, 8.30 p.m.: Clinical Meeting, at the Princess Elizabeth Orthopaedic Hospital.

### PLYMOUTH MEDICAL SOCIETY (Founded 1794) 1953–54 Session

Despite its antiquity the Society goes from strength to strength, and last season the numbers both of members and attendances at meetings exceeded all previous records. The Society still has the original minute books dating from its foundation in 1794. They show, among other things, the priority of discovery by Dr. E. L. Fox of the treatment of myxodema by thyroid gland in 1892. During the year the Library Committee of the B.M.A. cast covetous glances upon these books, but after a succinct debate the Society decided that they should remain in Plymouth, although loan for special purposes would be considered. The society also possesses two portraits of Edward Jenner; an original by Northcote was regarded by Jenner himself as the portrait which he liked best. It has recently been restored with the co-operation of the National Portrait Gallery. An excellent copy of this portrait was for many years owned by the Royal Albert Hospital, the Committee of which saved it from the cold hand of bureaucracy by presenting it to the Society in the early months of 1948. It continues therefore to adorn that part of the Hospital which so soon lost its royal title to become the Devonport "section", a designation which must have been inspired by some bureaucratic microtomist.

Last year's President provided an innovation by inviting Professor A. L. Rowse to give a mid-sessional address. His title was "Elizabethan England and Today"; his theme was the outburst of inspired activity which permeated England in the first Elizabethan reign and his belief that the present reign was about to witness a similar epoch in which discoveries of science, and among them those of medicine, would represent the discoveries of the Elizabethan adventurers. Speaking without a note, in a conversational style and with almost Gallic gestures, he carried us from the home life of a first Elizabethan lady through the achievements of the latter part of that reign to his vision of our not too distant future. It was a fascinating and inspiring address.

Perhaps it was hardly a coincidence that our next visiting speaker was Sir Russell Brain. It must be a rare, and probably an unique, event in the long life of our Society, to be addressed by the President of the Royal College of Physicians, and to hear an original exposition of the subject which, despite his many other activities, he had made his own. Cervical spondylosis became a reality in a way that no journal could make it.

Against this background other speakers might well feel that they flatulated against thunder. Far from it. Large and enthusiastic audiences heard Dr. Macdonald Critchley expose the intricacies of " The Body Image ", but it must be admitted that most of us, once separated from Dr. Critchley's persuasive diction, left this image to his academic care. Dr. P. M. F. Bishop's sound advice, backed by his researches, on " Endocrine Therapy " rapidly restored us to the fundamentals of practice, while our annual visit to the Royal Naval Hospital not only revealed the wealth of clinical problems encountered there but enabled us to hear at first hand of atomic explosions and of problems of high-altitude flying. In the face of all this talent our local boys made good. Great interest was aroused by Doctors Gilroy and Sweet on the " Psychological approach to Pruritus Ani ", by Dr. Jolly on " Hermaphrodites " and by Dr. Forbes on the " Treatment of Hemiplegia ". Three clinical evenings, rich and strange in their exhibits, completed the programme. Tigers as we are for work, our Annual dinner and dance was no less memorable than its predecessors. *Floreat aeternum.*

Dr. H. H. Cohen succeeds Mr. C. S. C. Prance as President for 1954–1955. Other officers are unchanged, with Mr. R. Howarth as Honorary Treasurer, Dr. C. R. Croft as Honorary Librarian, and Mr. W. B. Waterfall (3 Hartley Avenue, Plymouth) and Dr. J. W. N. Owen (Castlehayes, Plympton), as Honorary Secretaries.

### SOUTH SOMERSET MEDICAL CLUB

The South Somerset Medical Club membership is now approximately 50 and meetings of the Club are at present held on the third Thursday of the odd months of the year.

At the Annual General Meeting last year, Dr. S. Brimblecombe, Stoke-under-Ham, was elected President; Mr. J. A. Vere Nicholl, Vice-President, and Dr. P. P. Fox, Hon. Treasurer and Secretary.

A Clinical Meeting was held in November when Dr. Gardiner Hill of St. Thomas's Hospital gave an address on the Adrenal Glands.

In January there was a successful dinner and dance at which members invited guests.

In March Professor Milnes Walker gave an extremely interesting and instructive talk on Recent Advances in Cardiac Surgery which was illustrated by coloured films, and in May Dr. A. M. Best of Yeovil gave a very interesting talk on Medical Experiences as a Japanese P.O.W. in Malaya and Burma.

Forthcoming events include the Annual General Meeting which has been arranged for September. On November 18th Sir Arthur Porritt has agreed to come and address the Club, and it is hoped to arrange a joint meeting with the local branch of the B.M.A. for this occasion, and in January a Dinner and Dance will be held.

### TORQUAY AND DISTRICT MEDICAL SOCIETY

### Session 1953–54

The Society's activities have continued on a slightly reduced scale. Nine new members were elected during the Session, and the total membership is now 124.

Speakers during the Session were:

Dr. Alice Stewart: **" The Day to Day Work of a University Department of Social Medicine."**

Sir Zachary Cope: **" The Birth of Modern Scientific Medicine."**

Dr. G. H. Tovey: **" Salt and Water in Clinical Medicine."**

Dr. Meredith Davies: **" Is the Health Visitor really necessary? "**

Dr. Margaret Jackson: **" Investigations and Treatment of Subfertile Marriages with special reference to the Post-Coital Test."**

Professor Bruce Perry: **" Rheumatic Heart Disease."**

Dr. F. E. Camps: **" Death at Rillington Place."**

Dr. Hugh Jolly: **" Common Paediatric Disorders in General Practice."**

The average attendance at 6 Ordinary Meetings was 36 (30 last year). A Clinical Discussion on Health Visitors was attended by 20 members; the usual Clinical Meeting in conjunction with the S.W. Branch of the B.M.A. was again well attended, 50 signing the book.

A Medico-Legal subject was chosen for a joint meeting with Lawyers, Coroners, Dental Surgeons and Police; 112 were known to have been present. Dr. F. E. Camps spoke on the Christie murders at Rillington Place.

The Dinner and Dance after the Opening Meeting was again held at the Palace Hotel, and 120 members and guests were present.

A most interesting Summer Meeting was held at the Manor House, Torquay, including an address by the Warden on Rehabilitation of the Blind.

The Officers for 1954–55 are:

*President:* Dr. R. L. Midgley.

*Joint Hon. Secretaries:* Dr. I. R. Sutherland and Dr. C. P. Warren.

*Honorary Treasurer:* as before.

*Honorary Librarian:* as before.

### CORNWALL CLINICAL SOCIETY

The Annual Meeting of the Society was held at the Camborne—Redruth Hospital on Friday, 8th October, 1954, when the following Officers were elected for the coming year:

*President:* Mr. T. M. Banham, F.R.C.S., *Treasurer:* Dr. W. L. Stewart, *Secretaries:* Dr. J. D. Hardy, Dr. T. S. Wilson.

The retiring President, Dr. Pollock, gave an address on **"Glycosuria and Pregnancy".**

### B.M.A. BRISTOL DIVISION

### Clinical Meeting at Frenchay Hospital, Bristol

A Clinical Meeting was held by the Bristol Division of the B.M.A. in conjunction with the Branch, at Frenchay Hospital, on Wednesday, 16th June at 8 p.m. One hundred and eighty members and friends were present. In the absence of the Branch President, Professor Neale as Chairman of the Bristol Division presided.

Surgical cases were shown by members of the staff of the departments of neuro, thoracic, orthopaedic and general surgery and Dr. Pearson showed cases of chest disease. Mr. Jones of the University Veterinary Department, gave a demonstration of comparative medicine and Dr. Gaskell a series of X-ray films and techniques. An unusual, but welcome departure for a clinical meeting was an exhibition, arranged by Mr. Fitzgibbon, of ways and means of preventing burns and scalds.

Later, Mr. Alexander, Mr. Phillips, Mr. Belsey, Dr. Pearson, Mr. Routledge, Mr. Fitzgibbon, Mr. Jones and Dr. Gaskell presented the various cases demonstrated and under the stimulating Chairmanship of Professor Neale an interesting discussion was held.

In conclusion the Chairman thanked members of the hospital staff and in particular Mr. Bodenham who had done so much to make the meeting a great success.

R. C. Wofinden.

### Programme of Events 1954–55 Sessions

October 27th: Medical films, in Main Physics Lecture Theatre, Royal Fort.

November 24th: Dr. Harvey Flack, M.D., Editor of *Family Doctor:* " *Telling the Public about Medicine* " (B.M.A. Popular Lecture—members may invite a friend), in Main Physics Lecture Theatre, Royal Fort.

January 26th: Dr. Stewart Smith, M.A., M.D., Area Pathologist, Exeter and Mid-Devon Hospitals Management Committee, in Main Physics Lecture Theatre, Royal Fort.

Spring, 1955 (provisional): Social event—a sherry party to be arranged for members and friends.

May 18th (provisional): Annual General Meeting, in Main Physics Lecture Theatre, Royal Fort.

June 15th: Clinical Meeting at Southmead Hospital, Bristol.

June 24th, 4 p.m. (provisional): B.M.A. Reception for newly qualified doctors (Bristol University), Harden Hall, Royal Fort, Bristol.

### THE GLOUCESTERSHIRE BRANCH OF THE B.M.A.

The 1953–54 programme was brought to a close when the President (Dr. Logan) had as his guest Mr. Wylie McKissock, M.S., F.R.C.S., who delivered a stimulating address on the modern methods of investigation and treatment of cerebral vascular accidents. Mr. McKissock demonstrated, largely by the use of angiograms, how cerebral vascular accidents can be more accurately diagnosed now than in the past. Quite apart from the localization of haemorrhage occurring from an intracranial aneurysm he showed that many cerebral haemorrhages could be accurately localized and subsequently evacuated at operation, thereby removing what constituted a space-occupying lesion, with greatly improved changes of satisfactory functional recovery.

In the subsequent discussion it was evident that neurosurgical centres would be liable to inundation by patients suffering from cerebral vascular accidents if careful discrimination was not observed in the selection of patients for investigation. But Mr. McKissock laid stress on the fact that the results of treatment by surgery for cases of intracranial haemorrhage were so much better than those of treatment by the older conservative methods that Regional Boards would eventually be forced to increase the number of neurosurgical beds to cater for this new demand.

### Officers 1954–55

*President:* Dr. T. B. H. Haslett.
*President Elect:* Dr. H. S. K. Lowry.
*Vice President:* Dr. J. Greene (Senr.).
*Secretary:* Dr. E. W. Hyde.
*Assistant Secretary:* Dr. R. E. Bowers.

October 14th: President's Address (Dr. T. B. H. Haslett), "**Use and Abuse of Antibiotics**".
November 11th: **Clinical Meeting,** Gloucester Royal Infirmary.
December 9th: **Discussion on Pulmonary Tuberculosis.** Openers—Dr. F. J. Knights, Dr. R. H. Ellis, Dr. J. B. Hayward.
January 13th: To be arranged.
February 10th: Mr. J. F. Bourdillon, "**Osteopathic Methods in Orthopaedics**".
March 10th: **B.M.A. Lecture.**
April 14th: **Clinical Meeting,** Cheltenham General Hospital.
May 12th: Dr. R. E. Bowers, "**Disappointments in Dermatology**".

### WEST OF ENGLAND CHILD HEALTH GROUP

### Programme 1954–55

September 14th, 5.30 p.m. (Tea provided 4.45–5.30 p.m.): Mr. T. T. Stamm, F.R.C.S., Orth. Surg., Guy's Hospital: "**Recent Aspects of Foot Health.**" (This lecture is arranged in conjunction with Dr. Menzies Cormack, Divisional M.O.H., Gloucester County Council, and is part of the "Foot Health Week" programme for South Gloucestershire.) In the Main Physics Lecture Theatre, Royal Fort.

October 22nd, 5.30 p.m. R. C. Wofinden, M.D., D.P.H., Deputy M.O.H., Bristol: "**Health and Welfare Services in Sweden**": In the Children's Hospital.

November 11th, 7.45 p.m.: Members of the staff of the Children's Hospital. (N.B.—Cases on view 7.45–8.30 p.m. Discussion 8.30 onwards): **Clinical Demonstration on "The child who fails to thrive."** In the Children's Hospital.

December (date to be announced later), 8.30 p.m.: W. S. Craig, M.D., F.R.C.P.(E), F.R.S.(E), Professor of Child Health, Leeds University: **Subject to be announced later.** In the Main Physics Lecture Theatre, Royal Fort.

January 21st, 8.30 p.m.: Dr. Mary Sheridan, M.A., M.B., CH.B., D.C.H., Deputy M.O., Children's Department, Home Office: "**Speech and the Deaf Child**" (illustrated). In the Small Physics Lecture Theatre, Royal Fort.

February 25th, 5.30 p.m.: Mr. Vaughan-Davies, Music Adviser, Bristol Education Department: "**Music and the Child.**" In the Children's Hospital.

March 25th, 5.30 p.m. Dr. S. C. Walker, M.D., D.P.H., Senior M.O., M. and C.W., Bristol; Dr. S. Hinds, M.D., M.R.C.P., D.P.H., Lecturer in Child Health and Social Medicine; Miss E. Duncan, B.A., B.SC., Statistician, Health Dept.; Miss M. Reed, Welfare Officer, Health Department: "**Unmarried Parents.**" In the Children's Hospital.

April 29th, 8.30 p.m.: Dr. Grace Woods, M.B., B.S., D.P.H., D.C.H., Department of Child Health; Dr. H. M. Gibb, M.B., CH.B., D.P.H., Health Department; R. V. Saunders, M.A., B.ED., Educational Psychologist, Bristol: "**The Assessment of Cerebral Palsy**" (illustrated). In the Children's Hospital.

